# Medium-term Anatomical Results of Laser Peripheral Iridoplasty: An Anterior Segment Optical Coherence Tomography Study

**DOI:** 10.5005/jp-journals-10028-1235

**Published:** 2017-10-27

**Authors:** Joobin Hooshmand, James CY Leong, Jeremy O’Connor, Ghee S Ang, Anthony P Wells

**Affiliations:** 1 Consultant, Department of Ophthalmology, Royal Victorian Eye and Ear Hospital, East Melbourne, Victoria, Australia; 2 Registrar, Department of Ophthalmology, Capital & Coast District Health Board, Wellington, New Zealand; 3 Fellow, Department of Ophthalmology, Royal Victorian Eye and Ear Hospital, East Melbourne, Victoria, Australia; 4 Consultant, Department of Ophthalmology, Royal Victorian Eye and Ear Hospital, East Melbourne, Victoria, Australia; 5 Consultant, Department of Ophthalmology, Capital Eye Specialists Wellington, New Zealand

**Keywords:** Iridoplasty, Longitudinal, Optical coherence tomography, Primary angle-closure glaucoma.

## Abstract

**Aim:**

To evaluate by anterior segment optical coherence tomography (AS-OCT) the medium-term (mean duration 3.2 years) anatomical changes in the anterior chamber angle (ACA) after laser peripheral iridoplasty.

**Materials and methods:**

This is a longitudinal, retrospective case series of 31 eyes of 31 patients with primary angle-closure suspicion, primary angle closure (PAC), or primary angle-closure glaucoma (PACG) who underwent laser peripheral iridoplasty. All patients had persistent iridotrabecular contact (ITC) despite the presence of a patent peripheral iridotomy (PI). An AS-OCT was performed in dark conditions before and after laser iridoplasty. Quadrants of ITC, intraocular pressure (IOP), and the AS-OCT parameters of the temporal and nasal ACAs were measured and analyzed.

**Results:**

Prior to iridoplasty, the average number of quadrants of ITC was 3.3. At the first postiridoplasty visit (mean duration 6.8 weeks), this reduced to 1.7 quadrants but increased to 1.9 by the final follow-up visit (mean duration 3.2 years). Twenty-five patients (80.1%) had less ITC at the first postlaser visit increasing to 27 (87.1%) patients by the final visit. Two (6.5%) required a second iridoplasty, while 3 (9.7%) required cataract surgery. All parameters of angle width showed a statistically significant increase in magnitude. All patients maintained IOP ≥ 21 mm Hg throughout the follow-up period.

**Conclusion:**

Iridoplasty is a useful adjunct in widening the ACA, particularly in those with persistent angle closure after iridotomy but with no cataract. While not successful in all patients, it can act as a temporizing measure to widen the drainage angle until such time that cataract surgery can be performed.

**Clinical significance:**

Laser peripheral iridoplasty can be used as an adjunct in angle-closure glaucoma patients with no cataract.

**How to cite this article:** Hooshmand J, Leong JCY, O’Connor J, Ang GS, Wells AP. Medium-term Anatomical Results of Laser Peripheral Iridoplasty: An Anterior Segment Optical Coherence Tomography Study. J Curr Glaucoma Pract 2017;11(3):113-119.

## INTRODUCTION

Glaucoma is the leading cause of irreversible blindness worldwide.^1^ Despite its lower prevalence in comparison with primary open-angle glaucoma, PACG is responsible for a disproportionately large share of the visual morbidity attributable to glaucoma in Asian populations.^2^ The PACG is also a significant, and likely underdiagnosed, condition in Caucasian populations.^3^

Foster et al^4^ described standardized definitions of PACG along a spectrum of increasing severity from primary angle-closure suspect (PACS), PAC, and PACG.^5^ In the setting of PAC, the recommended initial nonsur-gical means of widening the ACA is by laser PI, which eliminates relative pupillary block.^5^ However, a proportion of eyes will still have residual angle closure, despite a successfully performed and patent iridotomy.^6^ In these eyes, particularly if they have no cataract, laser peripheral iridoplasty may be useful to treat persistent appositional angle closure occurring through non pupillary block mechanisms.^7,8^ Iridoplasty is thought to widen the ACA by thermal-induced contraction of the peripheral iris, as well as cross-sectional thinning of iris tissue.^9^ Current evidence suggests that iridoplasty is a useful adjunctive treatment tool for angle closure.^10^

Gonioscopy is the gold standard for assessment of ACA, but it can be highly subjective and observer depen-dent.^11^ Anterior segment optical coherence tomography allows objective, precise, and reproducible quantification of various anterior segment and angle anatomy param-eters.^12-14^ The AS-OCT is a noncontact imaging modality that rapidly obtains high-resolution cross-sectional images of the anterior segment with the patient seated upright. These features are advantageous compared with older imaging modalities, such as ultrasound biomicroscopy.^15^ Previous work by our group utilized AS-OCT to outline changes in anatomical features of the ACA in the short term following iridoplasty in a cohort of patients with persistent angle closure despite a patent iridotomy.^16^ Apart from a case series utilizing gonioscopy,^7^ there is a paucity of quantitative descriptions of the medium-term anatomical results of iridoplasty in the published literature. The aim of this study was to use AS-OCT to quantify the medium-term (mean duration 3.2 years) changes in ACA anatomy after iridoplasty in patients with residual angle closure despite a patent iridotomy.

## MATERIALS AND METHODS

This was a retrospective case series of 31 patients who underwent diode iridoplasty at Capital Eye Specialists, Wellington, New Zealand, recruited over a 41-month interval. All patients in this cohort had previously undergone laser iridotomy for PAC, PACS, or PACG, and had subsequent iridoplasty for persistent ITC despite a patent iridotomy.

Each newly referred patient with suspected angle closure received a full ocular examination. This included best-corrected Snellen visual acuity, slit-lamp evaluation, Goldmann applanation tonometry, corneal pachymetry, undilated fundoscopy, gonioscopy, and time domain AS-OCT imaging. The AS-OCT was performed by ophthalmic imaging technicians with the slit-lamp OCT (Heidelberg Engineering, GmBH, Dossenheim, Germany) in both uniform light and the dark with all room lights switched off. The scans were centered on the pupil.

The glaucoma specialist (A.P.W.) made the clinical decision as to whether the iridotomy was indicated based on AS-OCT and gonioscopy findings. The gonioscopy threshold for iridotomy was nonvisibility of the tra-becular meshwork in at least 180° of the ACA, consistent with the Association of International Glaucoma Societies consensus on angle-closure gonioscopy criteria.^17^ The AS-OCT threshold was extrapolated from these criteria as being the presence of ITC, visualized as apposition of peripheral iris to the inner corneoscleral wall anterior to the scleral spur, in at least two of four quadrants in dark conditions. In our study, this was defined to be consistent with PACS. The additional presence of peripheral anterior synechiae and/or IOP > 21 mm Hg distinguished PAC. The diagnosis of PACG was made if there were concurrent optic disk and visual field changes characteristic of glaucoma.

Laser PI was performed using the ophthalmic neodymium-doped yttrium-aluminum-garnet laser (Laserex Tango Nd:YAG, Ellex Medical, Australia) and an Abraham iridotomy contact lens. The PI was placed superiorly as close to the vertical meridian and as far peripherally as practical. Full-thickness perforation was confirmed by the gush of pigment and aqueous fluid from the posterior chamber into the anterior chamber. All iri-dotomies were confirmed patent in the study population.

The postiridotomy eyes with residual ITC in two or more quadrants in dark conditions as confirmed on repeat AS-OCT were deemed to still have occludable angles and were selected for iridoplasty. The mean time from iridotomy to iridoplasty was 34 weeks. Iridoplasty was performed using the Oculight SLx diode laser. Pupils were constricted with pilocarpine 2%. Thirty to thirty-five laser shots were applied on the iris as peripherally as possible over 360°, using a power between 200 and 350 mW. Maximum treatment time was 2.5 seconds, and spot size was 500 μm. The power and duration were titrated to be just enough to cause iris contraction but not superficial iris charring. After iridoplasty, patients were prescribed 5 days of topical prednisolone acetate 1.0% to relieve postlaser inflammation.

All patients received regular AS-OCT as well as routine clinical examination which included IOP measurement and gonioscopy at their follow-up visits. These visits were generally scheduled 1 to 2 months after iri-doplasty and every 6 months thereafter. The AS-OCT images from the final visit were reviewed and analyzed for each patient and compared with the images taken prior to iridoplasty. Although multiple scans of all four ACA quadrants were captured and treatment decisions were based on images from all four quadrants, only the horizontal images were analyzed in this study because these provided a clearer view of all ACA structures, in particular the scleral spur and peripheral iris recess, compared with the vertical images.

A single observer, a glaucoma fellow (G.S.A.), who was masked to the identity and sequence of the images being evaluated, measured the nasal and temporal quadrants for all eyes. The observer selected the best-quality image with clearly identifiable anatomical landmarks from a series of images of the nasal and temporal quadrants all centered on the pupil. Following the selection of the location of the scleral spur and iris recess apex, various anterior chamber drainage angle parameters were calculated using the slit-lamp OCT’s inbuilt analysis software including trabecular-iris angle (TIA), angle opening distance (AOD), trabecular-iris space area (TISA), and anterior chamber depth. Further parameters not included in the in-built analysis software were measured including trabecular-iris contact length (TICL), iris thickness (IT), and maximum iris bow height (MIBH). We had previously demonstrated moderate-to-good intraobserver reproducibility with the intraclass correlation coefficient statistic on all of these AS-OCT parameters in a larger cohort of patients using the same AS-OCT instrument and analysis software.^18^ Although the AS-OCT images were captured in both light and dark conditions, only results of scans in the dark were used for analysis.

**Figs 1A and B:**
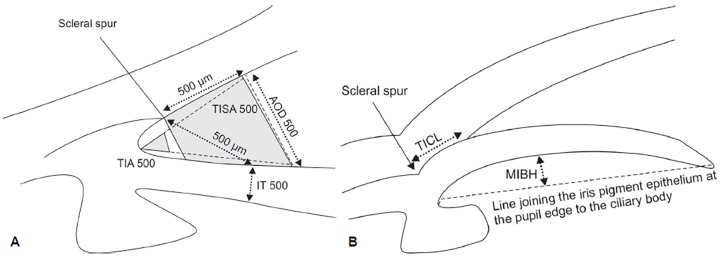
Anterior chamber drainage angle parameters measured with AS-OCT

Figure 1 summarizes the parameters measured with AS-OCT. The AOD 500 and 750 were the perpendicular distances from the trabecular meshwork at 500 and 750 μm anterior to the scleral spur to the anterior iris surface.^19^ The TIA 500 was considered the angle between the point of the trabecular meshwork 500 μm from the scleral spur and the point on the anterior iris perpendicularly, with the apex at the iris recess.^19^ The TIA 750 was similar to TIA 500 with the exception of the angle measured from the point of the trabecular meshwork 750 μm from the scleral spur. The TISA 500 was the trapezoidal area bordered anteriorly by the AOD 500, posteriorly by a line from the scleral spur perpendicular to the plane of the inner sclera to the anterior iris, superiorly by the inner corneoscleral wall, and inferiorly by the anterior iris surface.^20^ The TISA 750 was similar to TISA 500, except that it was bordered anteriorly by the AOD 750. The IT 500 was the perpendicular distance from the anterior iris surface at 500 μm from the scleral spur to the posterior iris pigment epithelial surface.^21^ The TICL was the length of contact between the anterior iris surface and the inner corneoscleral wall.^20^ The MIBH was used as a surrogate marker for iris curvature and was the perpendicular distance measured from the posterior iris pigment epithelial surface at its apex (the point where iris bowing was at its greatest) to the line joining the iris pigment epithelium at the pupil edge to its insertion at the ciliary body.^22^ The hyperreflective curve on the posterior iris surface marked the iris pigment epithelium; its insertion at the ciliary body was the point where the hyperreflective curve terminated within the ciliary body. Intraocular pressure was measured as an additional outcome.

Iridoplasty was defined as successful when ITC was observed in maximum of one quadrant, partially successful if ITC was observed in maximum of two quadrants, and failure if no improvement in ITC was observed and/ or cataract extraction was required to treat angle closure.

Data were analyzed using Microsoft Excel software. Basic descriptive statistics was conducted for patient demographics. Comparison of means was performed with the paired t-test for parametric data, while the Wilcoxon signed-rank test was used for nonparametric data. A p-value less than 0.05 was considered to be statistically significant. The study was conducted according to the tenets of the Declaration of Helsinki and had received approval from the Central Regional Ethics Committee of New Zealand.

Exclusion criteria were secondary angle closure, such as from uveitis, angle neovascularization and intumes-cent cataract, previous trauma, previous intraocular surgery, or poor-quality AS-OCT images that were unsuitable for angle evaluation. Only the right eye was used for analysis if both eyes were eligible.

## RESULTS

A total of 31 eyes of 31 patients were included in this study. The mean age at treatment with iridoplasty was 56.2 (±9.6) years. Twenty-nine patients (94%) were female. The majority of the patients were Caucasian (90%), with 2 being of Asian and 1 of Maori background. Four patients (13%) had PACG. The mean duration of follow-up after iridoplasty to the final follow-up visit was 38.6 months/3.2 years [2.4-71.2 months; standard deviation (SD) 20.5]. Four patients (12.9%) underwent cataract surgery, at a mean of 24 months postiridoplasty, and 3 (9.7%) due to persistent angle closure postiridoplasty. Two patients (6.5%) required redo-iridoplasty for persistent residual ITC, at a mean of 40 weeks postinitial Iridoplasty. All patients maintained IOP less than or equal to 21 at all follow-up clinic visits. Only the patients with confirmed glaucomatous damage (3 eyes) were on glaucoma treatment drops and only with a maximum of two agents.

Prior to iridoplasty, the average number of quadrants of ITC was 3.3. At the first postiridoplasty visit (mean duration 6.8 weeks), this reduced to 1.7 quadrants but increased to 1.9 by the final follow-up visit (mean duration 3.2 years). Twenty-five patients (80.1%) had less ITC at the first postlaser visit, increasing to 27 patients (87.1%) by the final visit.

At the first follow-up visit, the number of successful iridoplasties was 24 (77.4%), with 14 (45.1%) considered complete success and 10 (32.3%) partial success. This number changed to 8 (25.8%) completely successful iridoplasties and 18 (58.1%) partially successful cases by the end of the follow-up period. Overall 26 patients (83.9%) were successfully treated with iridoplasty at the final follow-up visit.

**Figs 2A to C:**
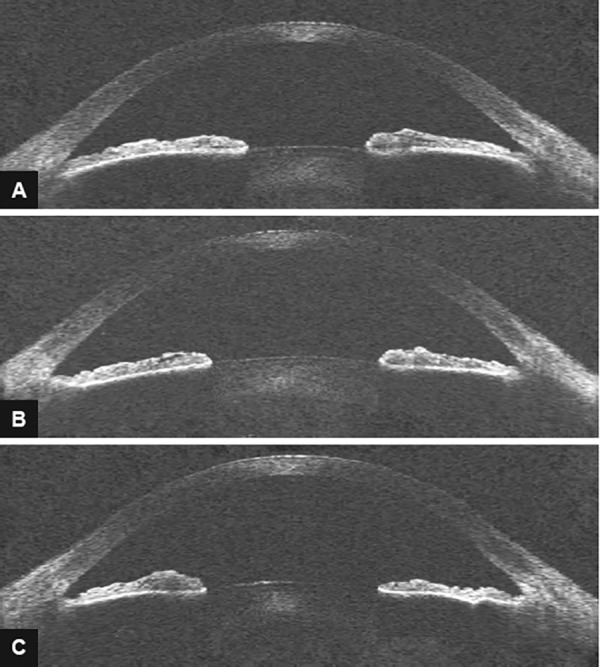
(A) Preiridoplasty AS-OCT appearance; (B) Postiridoplasty AS-OCT appearance at 5 weeks; and (C) Postiridoplasty AS-OCT appearance at 29.5 weeks

**Fig. 3:**
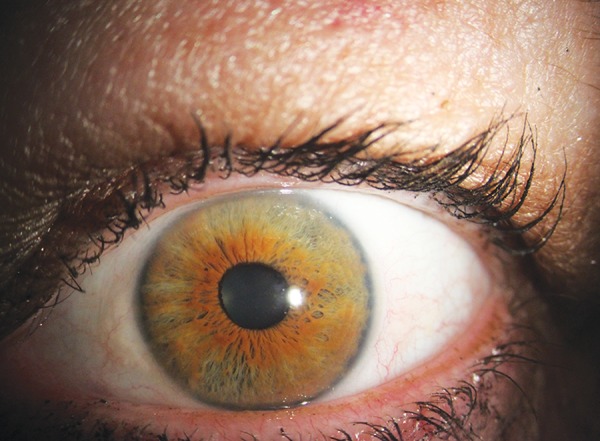
Typical appearance of postiridoplasty scars

After iridoplasty, all indicators of angle width (AOD 500, AOD 750, TIA 500, TIA 750, and TISA 500) showed a statistically significant increase in magnitude over the medium term. The IT 500 showed a statistically significant reduction in IT postiridoplasty. The TICL and MIBH both showed a statistically significant reduction in nasal and temporal angles. These parameters are summarized in Table 1. Typical AS-OCT changes pre- and postirido-plasty are shown in Figure 2. Typical postiridoplasty scar appearance is shown in Figure 3.

**Table 1:** **Table 1:** Changes in the anterior chamber and angle parameters postiridoplasty in the medium term

*Parameter*		*Mean preiridoplasty (SD)*		*Mean postiridoplasty (SD), μm*		*Mean diff (95% CI)*		p-value*	
AOD 500, μm									
Temporal		77 (79)		175 (92)		97 (64-130)		<0.0001	
Nasal		56 (61)		151 (74)		95 (63-127)		<0.0001	
TIA 500, degrees									
Temporal		8 (8)		18 (8)		10 (7-14)		<0.0001	
Nasal		6 (6)		16 (6)		10 (7-13)		<0.0001	
TISA 500, μm^2^									
Temporal		39 (30)		91 (71)		52 (26-78)		0.0003	
Nasal		23 (24)		65 (30)		42 (29-16)		<0.0001	
IT 500, μm									
Temporal		453 (97)		414 (62)		40 (1-78)		0.042	
Nasal		453 (63)**		393 (73)**		61 (30-92)		0.0004	
AOD750, μm									
Temporal		145 (103)		247 (115)		102 (58-146)		<0.0001	
Nasal		119 (82)		212 (92)		93 (12-62)		<0.0001	
TIA 750, degrees									
Temporal		10 (7)		16 (8)		6 (3-9)		<0.0001	
Nasal		9 (6)		15 (6)		6 (4-8)		<0.0001	
TISA 750, μm^2^									
Temporal		82 (52)		149 (83)		67 (33-101)		<0.0001	
Nasal		64 (39)		116 (43)		51 (32-71)		<0.0001	
TICL, μm									
Temporal		314 (272)		59 (139)		255 (158-350)		<0.0001	
Nasal		475 (196)		84 (13)		391 (293-489)		<0.0001	
MIBH, μm									
Temporal		137 (72)		90 (79)		47 ( 16-78)		0.0046	
Nasal		150 (77)		89 (77)		61 (34-88)		<0.0001	
ACD, mm		2.15 (0.34)		2.15 (0.30)				0.605	

V05; 31 eyes of 31 patients, mean follow-up of 39 months, *paired t-test, **only 30 patients; CI: Confidence interval

## DISCUSSION

Numerous AS-OCT studies have confirmed the changes in the ACA that occur after iridotomy.^18,23-25^ In the setting of persistent occludable angles after iridotomy, we previously documented that in the short term, additional iri-doplasty brings about significant widening of the ACA.^16^ This study does not report on gonioscopic findings as the aim was to quantify changes in angle configuration using AS-OCT.

We defined success as “complete” when ITC was observed in a maximum of one quadrant in dark conditions on AS-OCT, and hence, the eye was considered nonoccludable. A total of eight patients (25.8%) achieved complete success and were considered nonoccludable at their final follow-up visit. “Partial success” was when iridoplasty was able to reduce the number of quadrants of ITC on AS-OCT, but not to the extent that the eye could be considered completely nonoccludable (i.e., two residual quadrants of ITC on AS-OCT). Eighteen patients (58.1%) fell within this category. “Failure” occurred when irido-plasty did not reduce the number of quadrants of ITC on AS-OCT and/or when cataract extraction was required to treat persistent occludable angles. Cataract surgery is a known treatment option for angle-closure glaucoma.^26^ In our cohort, four patients underwent cataract surgery during the follow-up period, with three (3.12%) for persistent angle closure. Table 2 summarizes our success rates over the medium term.

**Table 2:** **Table 2:** Laser peripheral iridoplasty overall, complete, and partial success rate over the medium term

*Time*		*Failure (%)*		*Complete success (%)*		*Partial success (%)*	
Initial follow-up		22.5 (7/31)		45.1 (14/31)		32.3 (10/31)	
Final follow-up		16.1 (5/31)		25.8 (8/31)		58.1 (18/31)	

From our cohort of patients, the AS-OCT temporal and nasal angle parameters such AOD, TIA, and TISA, measured at both 500 and 750 μm from the scleral spur, showed a statistically significant increase 6 weeks after iridoplasty, and this was maintained over an average follow-up duration of 3 years. The IT 500 also showed a statistically significant reduction in peripheral IT in the medium term postiridoplasty.

Narayanaswamy et al^27^ in their randomized controlled trial (RCT) of 80 Asian patients with PAC or PACG also demonstrated similar increases of the indicators of the angle width, with AOD 500, AOD 750, TISA 750, and ARA 750 significantly increasing following iridoplasty at 1 year. However, the group went on to find that iri-doplasty on its own was inferior to medical therapy in maintaining IOP of less than 21 mm Hg at 1 year with an overall success rate of 70% using combined iridoplasty and medical therapy. Although our study did not specifically examine the effects of medical therapy, our cohort of patients maintained an IOP of 21 or less over the follow-up duration. A potential factor in the difference could be due to the patient population, with the RCT consisting of only Asians of predominantly Chinese background.

Iridoplasty has also been shown to reduce dependency on and reduce the number of antiglaucoma medications. In a prospective observation case-control study of 24 eyes in 12 patients, Ramakrishnan et al^28^ demonstrated a reduction by more than half in dependency and use of antiglaucoma medications at 1 year in patients who underwent iridoplasty.

Complications associated with iridoplasty are uncommon. One concern is the theoretical risk of corneal endothelial cell loss due to transfer of laser energy through the cornea. Muller et al^29^ could not demonstrate a significant difference in central endothelial cell density over an average of 30 months when comparing eyes that had undergone iridoplasty with the fellow untreated eye. Further, Narayanaswamy et al^27^ did not find any detrimental effect on the endothelial cell count or central corneal thickness at 1 year, while Ritch et al^7^ reported iridoplasty to be safe over a 6-year period with no complications from repeat treatment. Numerous other studies have also reported iridoplasty as safe with no significant associated complications.^16,25,28^

The limitations of our study include its retrospective design, single measure, and relatively small number of patients. We utilized scans only from the nasal and temporal quadrants due to the relative difficulty in identifying anatomical landmarks consistently in superior and inferior quadrants, a methodological issue that has previously been addressed in a similar fashion by other groups.^23,30,31^ It was also not possible to determine whether the AS-OCT scans imaged the exact same cross section of the angle before and after iridoplasty. We aimed to mitigate this by ensuring that all the AS-OCT images were centered on the pupil in the central horizontal meridian, in order to maximize scan consistency. A further limitation of AS-OCT is its inability to directly evaluate the ciliary body, such as for anterior rotation, which would have been of particular interest in this cohort of patients. Although this study utilized a single measurer, the scans being evaluated were fully masked, and the validity of the method already established as described above.

## CONCLUSION

This study describes systematically the changes observed by AS-OCT in the ACA configuration induced by irido-plasty in the medium term in a cohort of patients with residual angle closure after iridotomy. Our data suggest that iridoplasty remains a useful adjunct in medium-term widening of the ACA. This can be particularly helpful in those with persistent angle closure after iridotomy but with no cataract. Iridoplasty can therefore, act as a temporizing measure to widen the drainage angle until such time that cataract surgery can be performed.
